# Hazard Classification of Household Chemical Products in Korea according to the Globally Harmonized System of Classification and labeling of Chemicals

**DOI:** 10.1186/2052-4374-25-11

**Published:** 2013-07-16

**Authors:** Kyung-Hee Kim, Dae-Jong Song, Myeong-Hyun Yu, Yuon-Shin Park, Hye-Ran Noh, Hae-Joon Kim, Jae-Wook Choi

**Affiliations:** 1 Institute for Occupational and Environmental Health, Korea University, Seoul, Korea; 2 Graduate School of Public Health, Korea University, Seoul, Korea; 3 Department of Preventive Medicine, College of Medicine, Korea University College of Medicine, Seoul, Korea; 4 National Institute of Environmental Research, Incheon, Korea

**Keywords:** GHS, Consumer products, Household chemical products, Hazard communication

## Abstract

**Objectives:**

This study was conducted to review the validity of the need for the application of the Globally Harmonized System of Classification and Labeling of Chemicals (GHS) to household chemical products in Korea. The study also aimed to assess the severity of health and environmental hazards of household chemical products using the GHS.

**Methods:**

135 products were classified as ‘cleaning agents and polishing agents’ and 98 products were classified as ‘bleaches, disinfectants, and germicides.’ The current status of carcinogenic classification of GHS and carcinogenicity was examined for 272 chemical substances contained in household chemical products by selecting the top 11 products for each of the product categories. In addition, the degree of toxicity was assessed through analysis of whether the standard of the Republic of Korea’s regulations on household chemical products had been exceeded or not.

**Results:**

According to GHS health and environmental hazards, “acute toxicity (oral)” was found to be the highest for two product groups, ‘cleaning agents and polishing agents’, and ‘bleaches, disinfectants, and germicides’ (result of classification of 233 household chemical products) at 37.8% and 52.0% respectively. In an analysis of carcinogenicity assuming a threshold of IARC 2B for the substances in household chemical products, we found ‘cleaning agents and polishing agents’ to contain 12 chemical substances and ‘bleaches, disinfectants, and germicides’ 11 chemical substances.

**Conclusion:**

Some of the household chemical products were found to have a high hazard level including acute toxicity and germ cell mutagenicity, carcinogenicity, and reproductive toxicity. Establishing a hazard information delivery system including the application of GHS to household chemical products in Korea is urgent as well.

## Introduction

In the Republic of Korea, the hazards of household chemical products and providing information on these hazards has recently become an important issue, due to high profile cases of the death of a mother and infants using humidifier cleaner, wet tissues for baby use containing methylchloroisothiazolinone (2-methyl-4-isothiazolin-3-one, MIT), and the detection of carcinogens in outdoor clothing.

The Quality Control and Safety Management of Industrial Products Act regulates household chemical products. The act describes household chemical products most used by consumers that contain chemical hazards or may have hazards. The act regulates the following items only: cleaning agents, air fresheners, glues, polishing agents, deodorants, synthetic detergents, bleaches, and fabric softeners [1].

Korea’s policy on chemicals and chemical products management is addressed by the Toxic Chemicals Control Act of the Ministry of the Environment, the Quality Control and Safety Management of Industrial Products Act of the Ministry of Trade, Industry and Energy, and Material Safety Data Sheet (MSDS) regulations of the Ministry of Employment and Labor. However, the lack definitions for household chemical products and of a systematic policy for such products leaves consumers vulnerable. In short, regulations on hazards information and notice labeling for household chemical products is insufficient.

The Ministry of Trade, Industry and Energy partially revised section 41 of the Quality Control and Safety Management of Industrial Products Act in January 2012 [2]. The revision compels producers to submit data that include the ingredients, compounding ratio, and security-related information to the ministry. However, only a small proportion of the hazards information and caution labels are being provided to the public since companies are self-regulative and not compulsory.

The MSDS system is a way of providing safety information on chemicals. In Korea, importers and manufacturers of chemicals have been required to draft an MSDS for each chemical since 1996. It is composed of 16 sections (e.g. toxicological information, physical and chemical properties, etc.). Since the MSDS system is not applicable to general consumer merchandise, the safety control of household chemical products is not guaranteed by this approach.

The United Nations Conference on Environment and Development (UNCED) adopted the Globally Harmonized System of Classification and Labeling of Chemicals (GHS) in June 1992, with the objective of putting the GHS into use globally by 2008 [3-5].

GHS encompasses information on physical hazards (16 classes) and health and environmental hazards (11 classes) and systematizes hazard communication, including symbols, signal words, hazard statements, precautionary statements, and so on [6].

It is impossible to regulate every chemical by corresponding regulations; therefore, stipulating the form of notification will be effective to identify chemicals and to render information about hazards. Furthermore, the GHS applies to all hazardous chemicals. In the Republic of Korea, the GHS is applied to chemicals, regulated by the Ministry of Environment and Ministry of Employment and Labor [7]. It is difficult to regulate mixture substances according to each regulation. On the other hand, the GHS is applicable to mixture substances.

Providing hazard identification to the end users for household chemical products is the most reasonable and realistic alternative with respect to ensuring their right to know.

The studies on the GHS that have been performed and reported have mainly addressed the understanding level of workers and consumers concerning hazard communication about chemicals and the evaluation of effective national implementation of the GHS for each country [8-10].

However, a study examining the severity of hazards of chemical products by GHS classification has not been carried out. Therefore, in this study, the GHS classification for household chemical products was applied to inspect the severity of hazards. Moreover, to analyze the severity of chemical substances contained in household chemical products, we selected the substances most commonly contained in household chemical products and conducted a GHS classification.

Furthermore, we assessed the safety of whether the household chemical products are safer than the chemicals regulated by the Ministry of Employment and Labor and Ministry of Environment.

We also examined the severity of hazards, whether the limit, regulated by the Toxic Chemicals Control Act, was exceeded in order to establish policy support for building a system for providing hazards information for household chemical products.

## Materials and methods

### Subjects

We chose products according to the 2003 technical guidance document on risk assessment of the European Union (EU) for selecting and classifying the chemicals in use. The technical guidance document on risk assessment of the EU has 15 classes (cleaning agents, polishing agents, bleaches, germicides, disinfectants, adhesives and removers, etc.) and 96 sub-classes [11].

We selected household chemical products by visiting 4 major discount stores and studying the household chemical products for sale for 1 month in March 2009, April 2010, and May 2011. Since distribution through the internet is popular, we also selected 8 internet shopping malls and performed market research.

Among 15 classes of household chemical products, the product name, manufacturer, and distributor were identified for two classes: ‘cleaning agents and polishing agents’ and ‘bleaches, disinfectants and germicides’, which are chemicals commonly used in the home,. We requested the MSDS from each manufacturer and distributor and secured 155 products from ‘cleaning agents and polishing agents’ and 112 products from ‘bleaches, disinfectants and germicides’.

20 products from ‘cleaning agents and polishing agents’ and 14 from ‘bleaches, disinfectants and germicides’ were excluded because the MSDS information was not sufficient and the content of the ingredients was not printed.

The scope of this study is the following.

First, we selected 135 products from among the ‘cleaning agents and polishing agents’ and 98 products from the ‘bleaches, disinfectants and germicides’, and performed a GHS classification.

Second, to analyze the severity of hazards in the 272 chemical substances contained in the 233 household chemical products, a GHS classification was conducted for 22 chemical substances by selecting the top 11 chemical ingredients in each product.

Third, the carcinogenic classifications of the International Arctic Research Center (IARC) and American Conference of Governmental Industrial Hygienists (ACGIH) were investigated and analyzed for 272 chemical substances.

Fourth, to analyze the severity of hazards for the 233 household chemical products), we examined whether the limit of control standard was exceeded.

### Methods

#### Identification of ingredients of household chemical products

We requested the MSDS from the manufacturer and distributor and confirmed the Chemical Abstract Service (CAS) number included in the text of the MSDS, composition on ingredients, to identify chemical substances contained in household chemical products.

#### GHS classification principles

The GHS classification system, developed by the UN, categorizes hazards into physical hazards and health and environmental hazards [12]. The grade of hazards are classified with the numerical character of its category and the lower the grade, the greater the hazard. For instance, a ‘category 1’ acute toxicity is more hazardous than ‘category 5’.

The GHS classification methods are described as follows.

First, the ingredients are identified through the MSDS, written by the manufacturers of each household chemical product.

Second, the GHS is categorized by using 13 databases, which include the European Chemical Substances Information System (IUCLID) and IARC, and 4 reference works such as the Emergency Management Guides (ERG).

Third, the GHS classification of household chemical products proceeds after the classification of each chemical substance by applying the methods described in the mixture substance classification by the GHS, 3rd edition [6].

The GHS classification was conducted by using the Automatic GHS Classification Program for Mixture Substances, developed by researchers at Korea University. The credibility of the program was confirmed by 10 professionals. By classifying 300 chemical products manually, those who participated in the GHS classification project found there had been no classification errors.

#### GHS classification results for major chemical substances in household chemical products

We attempted to confirm the severity of hazards through the GHS classification on chemical substances contained in household chemical products. 135 products from the ‘cleaning agents and polishing agents’ were composed of 148 chemical substances and 98 products from the ‘bleaches, disinfectants and germicides’ were composed of 119 chemical substances.

We extracted the top 11 chemical substances, mostly contained from each product group, and conducted the GHS classification. Then the current carcinogen status, ruled by IARC, ACGIH, EU, and the National Toxicology Program (NTP), was investigated.

#### Hazardous severity assessment results on household chemical products (Related regulation applied)

To assess the hazards of 233 household chemical products, we examined whether the chemical substances regulated by Toxic Chemicals Control Act of the Ministry of Environment in Korea were used. When they were used, the severity of hazards was examined by checking whether it exceeded the limit of the standard restricted by the act.

## Results

### GHS classification result for each household chemical product

We classified the GHS health and environmental hazards for ‘cleaning agents and polishing agents’ and acute toxicity (oral) was the most common (37.8%, 51 products). This was followed by serious eye damage and irritation (31.8%, 43 products), and acute toxicity (inhalation; vapour) and being hazardous to the aquatic environment (chronic) (31.8%, 41 products,). In products that were carcinogenic, mutagenic, or had reproductive toxicity (CMR), the proportion of carcinogenicity was 6.7% (9 products), that of germ cell mutagenicity was 10.4% (14 products), and that of reproductive toxicity was 0.7% (1 product) (Table 1).

**Table 1:** **GHS hazard classification of** ‘**cleaning agents and polishing agents**’ (**n** = **135**)

	Hazard category^*^1	**Hazard category 2**	**Hazard category 3**	**Hazard category 4**	**Not classified**
Acute toxicity^†^					
Oral	1(0.7)	0(0.0)	6(4.5)	44(32.6)	84(62.2)
Dermal	0(0.0)	15(11.1)	9(6.7)	7(5.2)	104(77.0)
Inhalation (gases)	0(0.0)	0(0.0)	1(0.7)	2(1.5)	132(97.8)
cInhalqation (vapours)	1(0.7)	16(11.9)	23(17.1)	1(0.7)	94(69.6)
Inhalation (dust/mist)	0(0.0)	1(0.7)	6(4.5)	1(0.7)	127(94.1)
Skin corrosion/irritation^‡^	17(12.6)	13(9.6)	-	-	105(77.8)
Serious eye damage/irritation^‡^	23(17.0)	20(14.8)	-	-	92(68.2)
Respiratory sensitization^‡^	6(4.4)	-	-	-	129(95.6)
Skin sensitization^‡^	6(4.4)	-	-	-	129(95.6)
Germ cell mutagenicity^‡^	14(10.4)	0(0.0)	-	-	121(89.6)
Carcinogenicity^‡^	9(6.7)	0(0.0)	-	-	126(93.3)
Reproductive toxicity^‡^	1(0.7)	0(0.0)	-	-	134(99.3)
Specific target organ toxicity^‡^					
Single exposure	0(0.0)	1(0.7)	2(1.5)	-	132(97.8)
Repeated exposure	0(0.0)	1(0.7)	-	-	134(99.3)
Aspiration hazard^‡^	18(13.3)	0(0.0)	-	-	117(86.7)
Hazardous to the aquatic environment (acute)^‡^	7(5.2)	-	-	-	128 (94.8)
Hazardous to the aquatic environment (chronic)^‡^	1(0.7)	18(13.3)	11(8.2)	11(8.2)	94(69.6)

N (%).

*It is labeled as “Danger” or “Warning” in accordance with the division of each of the hazard classification degrees of toxicity, and it is labeled as “Danger” if the degree of toxicity of the hazard is high.

^†^Regarding the acute toxicity, if the classification is hazard category 1, hazard category 2, and hazard category 3, then the warning statement indicates “Danger”. 7 products with acute toxicity (oral), 24 products with acute toxicity (dermal), and 48 products with acute toxicity (inhalation) were categorized as requiring “Danger” warning statements.

^‡^17 products causing skin corrosion/irritation, 23 products causing serious eye damage/irritation, 6 products causing respiratory sensitization, 6 causing skin sensitization, 14 causing germ cell mutagenicity, 9 products causing carcinogenicity, 1 product causing reproductive toxicity, 18 products causing an aspiration hazard, and 7 products hazardous to the aquatic environment (acute) were all classified as GHS hazard classification result hazard category and the ‘warning statement’ of “Danger” was stipulated.

From the results of the GHS health and environmental hazards toxicity analysis of the ‘bleaches, disinfectants and germicides’, the acute toxicity (oral) was the most common (52.0%, 51 products). This was followed by serious eye damage and irritation (50.0%, 49 products), and then by acute toxicity (inhalation; vapour) (33.7%, 33 products). In the CMR products, the proportion that were carcinogenic was 15.3% (15 products), of germ cell mutagenicity was 3.1% (3 products), and that of reproductive toxicity was not categorized (Table 2).

**Table 2:** **GHS hazard classification of** ‘**bleaches**, **disinfectants and germicides**’ (**n** = **98**)

	Hazard category^* ^**1**	**Hazard category 2**	**Hazard category 3**	**Hazard category 4**	**Not classified**
Acute toxicity^†^					
Oral	0(0.0)	0(0.0)	3(3.0)	48(49.0)	47(48.0)
Dermal	0(0.0)	4(4.1)	9(9.2)	11(11.2)	74(75.5)
Inhalation (gases)	4(4.1)	0(0.0)	0(0.0)	1(1.0)	93(94.9)
Inhalation (vapours)	3(3.1)	9(9.2)	11(11.2)	10(10.2)	65(66.3)
Inhalation (dust/mist)	0(0.0)	0(0.0)	1(1.0)	5(5.1)	92(93.9)
Skin corrosion/irritation^‡^	15(15.3)	11(11.2)	-	-	72(73.5)
Serious eye damage/irritation^‡^	20(20.4)	29(29.6)	-	-	49(50.0)
Respiratory sensitization^‡^	2(2.0)	-	-	-	96(98.0)
Skin sensitization^‡^	6(6.12)	-	-	-	92(93.9)
Germ cell mutagenicity^‡^	3(3.1)	0(0.0)	-	-	95(96.9)
Carcinogenicity^‡^	15(15.3)	0(0.0)	-	-	83(84.7)
Reproductive toxicity^‡^	0(0.0)	0(0.0)	-	-	98(1.00)
Specific target organ toxicity^‡^					
Single exposure	0(0.0)	0(0.00)	4(4.1)	-	94(95.9)
Repeated exposure	0(0.0)	4(4.1)	-	-	94(95.9)
Aspiration hazard^‡^	6(6.1)	0(0.0)	-	-	92(93.9)
Hazardous to the aquatic environment (acute)^‡^	23(23.5)	-	-	-	75(76.5)
Hazardous to the aquatic environment (chronic)^‡^	5(5.1)	11(11.2)	3(3.1)	0(0.0)	79(80.6)

N (%).

^*^It is labeled as “Danger” or “Warning” in accordance with the division of each of the hazard classification degrees of toxicity, and it is labeled as “Danger” if the degree of toxicity of hazard is high.

†Regarding acute toxicity, if the classification is hazard category 1, hazard category 2 and hazard category 3, then the warning statement is “Danger”. 3 products having acute toxicity (oral), 13 products with acute toxicity (dermal), and 28 products with acute toxicity (inhalation) were categorized to be indicated with “Danger” warning statements.

^‡^15 products causing skin corrosion/irritation, 20 products causing serious eye damage/irritation, 2 products causing respiratory sensitization, 6 products causing skin sensitization, 3 products causing germ cell mutagenicity, 15 products with carcinogenicity, 6 products causing aspiration hazard, and 23 products hazardous to the aquatic environment (acute) were classified by the GHS hazard classification in the hazard category and the ‘warning statements’ was indicated to be “Danger”.

### GHS classification results on the main chemical substances in household chemical products

The top 11 chemical substances were selected from 135 products in the ‘cleaning agents and polishing agents’ class and 98 products from the ‘bleaches, disinfectants and germicides’. For 22 chemical substances, the GHS classification results were the following.

### GHS classification for the main ingredients in ‘cleaning agents and polishing agents’

The most commonly used chemical substances in the ‘cleaning agents and polishing agents’ were sodium carbonate (CAS No. 497-19-8) and sodium laureth sulfate (CAS No. 9004-82-4). They were used in 16 out of 135 products. This was followed by sodium alpha-olefin (c14-c16. sulfonate, CAS No. 68439-57-6) in 14 products, isoparaffinic hydrocarbon (CAS No. 64742-48-9) in 12 products, and ethoxylated alcohols (c12-c15) (CAS No. 68551-12-2) and poyloxyethylene (CAS No. 9002-92-0) each used in 11 products. Sodium carbonate, the most frequently used ingredient, was classified as acute toxicity (dermal) ‘Category 2’, acute toxicity inhalation (vapour) ‘Category 3’, serious eye damage and irritation ‘Category 2’. Isoparaffinic hydrocarbon was classified in acute toxicity inhalation (vapour), germ cell mutagenicity, and hazardous to the aquatic environment (chronic) by the GHS classification (Table 3).

**Table 3:** **GHS hazard classification results on ingredients contained in multiple products from the household** ‘**cleaning agents and polishing agents**’ **product group**

**Order**	**Products using the chemical**	**CAS no.**	**Chemical name**	**Hazard class and category**	**Signal word**	**Hazard statement**
1	16	497-19-8	Sodium carbonate	Acute toxicity (dermal)-cat. 2	Danger	Fatal in contact with skin
				Acute toxicity (vapours)-cat. 3	Warning	Toxic if inhaled
				Eye damage /Irritation-cat. 2		Causes serious eye irritation
1	16	9004-82-4	Sodium laureth sulfate	Acute toxicity (oral) -cat. 4	Warning	Harmful if swallowed
3	14	68439-57-6	Sodium alpha-olefin (c14-c16) sulfonate	·Not classified	-	-
4	12	64742-48-9	Isoparaffinic hydrocarbon	Acute toxicity (vapours)-cat. 4	Danger	Harmful if inhaled
				environment (chronic)-cat. 4	Warning	May cause genetic defects
				Germ cell mutagenicity-cat. 1B		May cause cancer
				Aspiration hazard-cat. 1		May be fatal if swallowed and enters airways
				Hazardous to the aquatic		May cause long lasting harmful effects to aquatic life
4	11	68551-12-2	C12-c15)/(ethoxylated alcohols (c12-c15)	Not classified	-	-
6	11	9002-92-0	Poyloxyethylene	Acute toxicity (oral)-cat. 4	Danger	Harmful if swallowed
7	10	56-81-5	Glycerine	Acute toxicity (vapours)-cat. 2	Danger	Fatal if inhaled
7	10	9014-01-1	Subtilisins	Skin corrosion/irritation-cat. 2	Danger	Causes skin irritation·
				Eye damage /Irritation-cat. 1	Warning	Causes serious eye damage
				Respiratory sensitization-cat. 1		May cause allergy or asthma symptoms or breathing difficulties if inhaled
				Specific target organ toxicity (single exposure)-cat. 3		May cause respiratory irritation
9	9	61789-40-0	Coconut oil amidopropyl betaine	Hazardous to the aquatic environment (acute)-cat. 1	Warning	Very toxic to aquatic life
9	9	1643-20-5	N,n-dimethyldodecylamine n-oxide	Hazardous to the aquatic environment (chronic)-cat. 2	-	May cause long lasting harmful effects to aquatic life
9	9	27176-87-0	Dodecylbenzenesulfonic acid	Acute toxicity (oral) -cat. 4	Warning	Harmful if swallowed

### GHS classification for the main ingredients in ‘bleaches, disinfectants and germicides’

The most commonly used chemical in the ‘bleaches, disinfectants and germicides’ was ethyl alcohol (CAS No. 64-17-5), which was in 29 out of 98 products. This was followed by sodium hydroxide (CAS No. 1310-73-2) was in 13 products, sodium hypochlorite (CAS No. 7681-52-9) in 12 products, isopropyl alcohol (CAS No. 67-63-0) in 9 products, and 5-chloro-2-methyl-4-isothiazolin-3-one (CAS No. 26172-55-4) in 8 products.

Ethyl alcohol, the most commonly used chemical in the ‘bleaches, disinfectants and germicides’, was classified as ‘category 4: acute toxicity (oral)’ and ‘category 1A: carcinogenicity’. Sodium hydroxide was classified as ‘category 4: acute toxicity (oral)’ and ‘category 1 (danger): skin corrosion and irritation’, and sodium hydroxide contained lead and arsenic, classified as having CMR toxicity. The group of bleaches was confirmed to contain lead and arsenic and the MSDS for each product reported that the two metals had a concentration of less than 1 ppm (Table 4).

**Table 4:** **GHS hazard classification result on ingredients contained in multiple products of the household** ‘**bleaches**, **disinfectants and germicides**’ **product group**

**Order**	**Products using the chemical**	**CAS no.**	**Chemical name**	**Hazard class and category**	**Signal word**	**Hazard statement**
1	29	64-17-5	Ethyl alcohol	Acute toxicity (oral)-cat. 4	Warning	Harmful if swallowed
				Germ cell mutagenicity-cat. 1A	Danger	May cause cancer
2	13	1310-73-2	Sodium hydroxide	Acute toxicity (oral)-cat. 4	Danger	Harmful if swallowed
				Skin corrosion/irritation-cat. 1	Warning	Causes severe skin burns and eye damage
3	12	7681-52-9	Sodium hypochlorite	Acute toxicity (vapours) -cat. 4	Danger	Very toxic to aquatic life
				Skin corrosion/irritation-cat. 1	Warning	Causes severe skin burns and eye damage
				Hazardous to the aquatic environment-cat. 1		
4	9	67-63-0	Isopropyl alcohol	·Eye damage/irritation-cat. 2·	Warning	Causes serious eye damage·
				Specific target organ toxicity (single exposure)-cat. 3		May cause respiratory irritation
5	8	26172-55-4	5-chloro-2-methyl-4-isothiazolin-3-one	Acute toxicity (oral)-cat. 4	Danger	Harmful if swallowed
				Acute toxicity (dermal) -cat. 4	Warning	May cause allergy or asthma symptom or breathing difficulties if inhaled
				Acute toxicity (vapours) -cat. 2		Very toxic to aquatic life
				Hazardous to the aquatic environment (acute)-cat. 1		
				Hazardous to the aquatic environment (chronic)-cat. 1		
6	7	15630-89-4	Sodium percarbonate	Not classified	-	-
6	7	497-19-8	Sodium carbonate	Acute toxicity (dermal)-cat. 2	Danger	Fatal in contact with skin
				Acute toxicity inhalation (dust/mist)-cat. 3	Warning	Toxic if inhaled
				Eye damage /Irritation-cat. 2		Causes serious eye damage
6	7	64742-47-8	hydrotreated light distillate	Acute toxicity (vapours)-cat. 2	Danger	Toxic if inhaled
				Aspiration toxicity-cat. 1		May be fatal if swallowed and enters airways
				Hazardous to the aquatic environment (chronic)-cat. 2		May cause long lasting harmful effects to aquatic life
6	7	7439-92-1	Lead	Hazardous to the aquatic environment (acute)-cat. 1·	Warning	Very toxic to aquatic life·
				Germ cell mutagenicity-cat. 2		May cause cancer
6	7	7440-38-2	Arsenic	Acute toxicity (oral)-cat. 4·	Danger	Harmful if swallowed·
				Carcinogenicity-cat. 1A	Warning	May cause cancer
6	7	7440-43-9	Cadmium	Acute toxicity inhalation (dust/mist)-cat. 1·	Danger	Fatal if inhaled·
				Germ cell mutagenicity-cat. 2	Warning	Suspected of causing genetic defects
				Carcinogenicity-cat. 1A		May cause cancer
				Specific target organ toxicity (repeated exposure)-cat. 1		Causes damage to organs through prolonged or repeated exposure
				Hazardous to the aquatic environment (acute)-cat. 1		Very toxic to aquatic life

### Carcinogenicity classification results on the ingredients in household chemical products and the results of applying the Toxic Chemicals Control Act (TCCA)

A carcinogenicity classification was conducted for 121 chemical substances in the ‘cleaning agents and polishing agents’, and 151 chemical substances in ‘bleaches, disinfectants and germicides’. The product group of ‘cleaning agents and polishing agents’ contained 4 chemical substances classified higher than 2B from the IARC carcinogen categories and that of ‘bleaches, disinfectants and germicides’ contained 6 such chemical substances. 1 product for ‘cleaning agents and polishing agents’ and 5 products for ‘bleaches, disinfectants and germicides’ contained chemical substances classified as A1 (confirmed human carcinogen) and A2 (suspected human carcinogen) (Table 5).

**Table 5:** Carcinogenicity classification on the ingredients in household chemical products

**Carcinogenicity**	**Cleaning agents and polishing agents**	**Bleaches, disinfectants and germicides**	**Total**
IARC^*^			
Group 1	2	5	2
Group 2A	0	0	0
Group 2B	2	1	2
Group 3	8	11	8
ACGIH^†^			
A1	0	2^∏^	0
A2	1	3	1
A3	1	2	1
A4	5	4	5
EU^‡^			
Category 1	3	3	3
Category 2	5	2	5
Category 3	0	1	0
NTP^§^			
R	0	1	0
K	1	4	1

(N = 272 chemical substances)

^*^International Agency for Research on Cancer (IARC).

Group 1: carcinogenic to humans, Group 2A: probably carcinogenic to humans, Group 2B: possibly carcinogenic to humans, Group 3: not classifiable as to carcinogenicity to humans, Group 4: probably not carcinogenic to humans.

^†^American Conference of Governmental Industrial Hygienists (ACGIH).

A1: confirmed human carcinogen, A2: suspected human carcinogen, A3: animal carcinogen, A4: Not classifiable as a human carcinogen.

^‡^European Union, EU.

Category 1: Substances known to be carcinogenic to humans. Category 2: Substances which should be regarded as if they are carcinogenic to humans. Category 3: Substances which cause concern for humans, owing to possible carcinogenic effects but in respect of which the available information is not adequate for making a satisfactory assessment.

^§^National Toxicology Program (NTP).

*K* known to be human carcinogens.

*R*reasonably anticipated to be human carcinogens.

^∏^CAS no. 7440-47-3. Overlapping of ACGIH A1 and A4 as a chemical substance that belongs to salts and chemical products.

### The result of toxic severity of household chemical products: application of regulations for chemicals

Products exceeding the limits for each category (poisonous substances, substances requiring preparation for accidents, restricted or prohibited substances; regarding the chemical substances in chemical products), regulated by the Toxic Chemicals Control Act, was investigated to assess the severity of health and environmental hazards. Since the threshold for toxicity of cleaning agents and synthetic detergents differs between the standard of the Ministry of Trade, Industry and Energy and the Toxic Chemicals Control Act, which this study adopted, the standard of the Ministry of Trade, Industry and Energy was excluded. 13 chemical substances, restricted by the Toxic Chemicals Control Act, were used in 23 chemical products in the ‘cleaning agents and polishing agents’ and 8 products exceeded the limit. 16 chemical substances restricted by the Toxic Chemicals Control Act were used in 29 household chemical products in the ‘bleaches, disinfectants and germicides’ and 4 products exceeded the limit (Table 6).

**Table 6:** Usage of toxic chemicals in household chemical products

	**Number of products that containing toxic chemicals**	**Number of products that exceeding the allowed limit**
Cleaning agents and polishing agents (N = 135)	23(17.0%)	8(5.9%)^*^
Bleaches, disinfectants and germicides (N = 98)	29(29.6%)	4(4.1%)^†^

^*^There were 8 products among the ‘cleaning agents and polishing agents’ that used substances regulated by the Toxic Chemical Control Act in excess of the allowed value including 5 products that ambiguously indicated the contents under the MSDS.

^†^There were 4 products among the ‘bleaches, disinfectants and germicides’ that used substances regulated by the Toxic Chemical Control Act in excess of the allowed value including 2 products for which the legal regulations on the corresponding products were difficult to determine.

## Discussion and conclusions

This study was conducted to confirm the hazards of mixture substances (unregulated, since regulations center on chemical substances) and to provide the basis for establishing a regulatory policy for mixture substances.

The objects of this study were 233 products, after 34 products (13.0%) were excluded from 267 products in the two classes of ‘cleaning agents and polishing agents’ and ‘bleaches, disinfectants and germicides’. The reason for their exclusion was that the composition and information on ingredients were not written in the MSDS and the CAS number was not accurate. The hazards can be confirmed only if the ingredients are identified, so it is dependent on the MSDS provided from the manufacturing company. However, corporations have not disclosed the information, using the excuse of confidentiality, and the composition and information on ingredients have either been not written or partially written [13,14].

Lee (2009) reported that the ingredients could not be identified for about 45.0% of mixture substances in the MSDS [15]. As described above, if the ingredients information in the MSDS is not accurate, the establishment of Precautionary Principle hazards communication will be difficult.

European Chemicals Agency (ECHA) regulates CMR substances as Substances of Very High Concern (SVHC) [16] 20 products (13.5%) from the ‘cleaning agents and polishing agents’ and 7 products (7.0%) from the ‘bleaches, disinfectants and germicides’ were classified as CMR substances. Such CMR substances could not be investigated accurately and the applicability of each regulation was not fully determined; therefore, the potential for being exposed to toxicity is very high [17]. Lee (2011) reported additional information for applying the UN GHS standard to CMR chemical substances is needed to improve the industrial health and awareness of carcinogenicity, since the standard of classification and labelling method are used differently [18].

When analyzed by the IARC, ACGIH, EU, and NTP, 7 products contained chemicals classified as carcinogenic by the IARC for ‘cleaning agents and polishing agents’, and 4 products contained chemicals categorized as carcinogenic by the IARC for the ‘bleaches, disinfectants and germicides’ such chemicals. The main products which used the chemicals categorized as carcinogenic, were polishes for cars, ingrained stain removers, and multi-purpose cleaning agents in ‘cleaning agents and polishing agents’.

From the ‘bleaches, disinfectants and germicides’, the main products include pesticides, bactericides, and bleaches for cleaning. 29 chemical substances were categorized as carcinogenic by the IARC among 272 chemical substances which compose the product groups ‘cleaning agents and polishing agents’ and ‘bleaches, disinfectants and germicides’; 18 chemical substances are carcinogenic by the ACGIH; 14 by the EU; and 6 by the NTP.

In the systems of the Quality Control and Safety Management of Industrial Products Act, Occupational Safety and Health Act, and Toxic Chemicals Control Act, there is no specific clause regarding carcinogenicity. The first condition for restricting manufacture is in the case of carcinogenicity. Subsequent provisions for permitting manufacturing or restricting handling by the Toxic Chemicals Control Act can be applied [19].

The excess of limits for each category (poisonous substances, substances requiring preparation for accidents, restricted or prohibited substances; regarding single substances in household chemical products), regulated by Toxic Chemicals Control Act, was investigated.

To assess the severity of health and environmental hazards, a study was conducted regarding whether poisonous substances, substances requiring preparation for accidents, and restricted or prohibited substances (categorized by the Toxic Chemicals Control Act of the Ministry of Environment) are regulated. The result of that study showed that 8 products from among the ‘cleaning agents and polishing agents’ and 4 products from the ‘bleaches, disinfectants and germicides’ exceeded the limits of regulation. Based on the findings, these household chemical products are not safe to use.

From the results of this study, the toxicity, including CMR and acute toxicity, was high for a portion of household chemical products. The introduction of communication of chemical hazards information (such as including a caution mark on household chemical products) and establishment of regulations are urgently required.

The GHS warns with caution marks, including symbols and signal words. Such caution marks will be effective ways to warn of hazards and to reduce damages from careless handling. Kim and You (2000) reported that the purpose of a caution mark is to influence the attitudes of consumers with respect to the hazard and providing the caution mark will be an excellent method for effectively influencing their attitude [20]. The attachment of caution marks or signs according to GHS classification will be meaningful since those can influence consumers effectively.

The fact that areas like the EU and Thailand have applied the GHS to household chemical products to render chemical hazards information more easily shows that there are important implications for preventive measures for health.

Countries in the EU are replacing the regulations on chemical products with classification, labeling and packaging (CLP) systems [21]. A CLP is similar to the classification system of the GHS, and every imported product should be classified and labeled and packaged according to the CLP. The United States regulates products by the Federal Hazardous Substances Act (FHSA) of the Consumer Product Safety Commission (CPSC). The CPSC (2008) completed a comparison analysis for of the GHS and FHSA and decided that an alteration of the FHSA is needed [22].

Canada regulates products with the Consumer Chemicals and Containers Regulation. The country does not apply the GHS, but the country’s regulations have adopted labeling [23]. Furthermore, the United States, Canada, and the EU have enacted laws regarding toxic chemical containing products, and have required marking of different types of hazards (e.g. toxic, corrosive, irritative, etc.). Moreover, visual notation including signs and pictures must be used to render the marks of caution according to the law [24].

As of February, 2012, Thailand, Malaysia, and Australia are discussing introducing the GHS for household chemical products and the Food and Drug Administration (FDA) in Thailand has completed an analysis of the validity of applying the GHS to chemical products and is proceeding to obtain the approval of the Health Minister [25]. The Department of Health and Ageing (DoHA) in Australia is conducting a study on introducing the GHS with other agencies by publish a discussion paper [26,27]. In Switzerland, classification and labelling with the GHS standard is slated for implementation by December 1, 2012 and revising regulations on mixtures by June 1, 2015 [27]. In the Republic of Korea, there are no regulations for adding a caution mark to chemical products, but the safety certification system by Quality Control and Safety Management of Industrial Products Act is under preparation [1].

Countries in EU require that the manufacturer register an MSDS in the Products Register and the registered MSDS provides information for medical staff through the Poison Center and renders emergency treatment information. Austria has required that the MSDS of new chemical products be registered with the Federal Environment Agency, and Switzerland requires that every chemical product be registered with the Chemical Products Division of the Swiss Federal Office of Public Health. The National Library of Medicine (NLM) opens MSDS records to the public [28]. This allows for perusing the ingredients and content (%) of specific chemical products and chemical hazards information.

In Korea, only safety certification status and the product’s image are provided through the website of the Korean Agency for Technology and Standards in the Ministry of Trade, Industry and Energy [29]. The Ministry of Environment revised its regulations on toxic chemicals control to found legal basis and has operated the Chemical Accident Emergency Information System (CEIS) since 2009.

The CEIS has 2,400 types of hazards information on chemicals and household chemical products and opens the GHS classification to the public through its website [30-32]. The problem of multiple regulations on providing cautionary information by the Toxic Chemicals Control Act, Quality Control and Safety Management of Industrial Products Act, and Occupational Safety and Health Act may occur.

Furthermore, application of the GHS to household chemical products has problems such as the costs to companies and the training of professionals to perform classification. For that reason, further discussion of the ripple effects of introducing the GHS is needed. However, the GHS is applicable to every chemical substance and mixture substance and has standards of classification and renders easier understanding by using cautions and labels. The value of introducing the GHS is clear with respect to preventive health.

A limitation of this study is that the GHS classification was conducted on the basis of chemicals and CAS number, written on the MSDS, provided by companies. If the CAS number on the MSDS was reported incorrectly, the GHS classification results may be incorrect as well. However, we confirmed the agreement of the CAS number and the name of the product. When necessary, we contacted the manufacturer to clarify.

In this study, we were able to confirm the severity of hazards through the GHS for household chemical products, and this will be helpful in establishing a strategy for introducing the GHS.

## Competing interests

The authors have declared that no competing interests exist.

## Author’s contributions

JW and HJ took charge of the overall planning and evaluation of this paper. KH carried out the overall evaluation for the GHS hazard classification of household chemical products and the analysis for the political trend of the studies related with the hazards of household chemical products. DJ and MH participated in the GHS hazard classification of household chemical products and chemical substance, and HR and YS participated in the carcinogenicity classification of household chemical products. All authors read and approved the final manuscript.
